# Perioperative Anesthesia Precautions in Oral and Maxillofacial Surgery During the COVID-19 Pandemic: A Practical Review

**DOI:** 10.5152/eurasianjmed.2022.20408

**Published:** 2022-02-01

**Authors:** Dilek Günay Canpolat

**Affiliations:** Department of Oral and Maxiloofasial Surgery, Erciyes University School of Dentistry, Kayseri, Turkey

**Keywords:** Anesthesia management, COVID-19, maxillofacial surgery

## Abstract

The coronavirus disease 2019 has been defined as COVID-19 in February 2020 by the World Health Organization. It rapidly resulted in an epidemic throughout China and spread over the world causing a pandemic. Anesthetic procedures such as tracheal intubation, non-invasive ventilation, tracheotomy, cardiopulmonary resuscitation, manual balloon-mask ventilation before the intubation, and bronchoscopy in operating rooms are aerosol-generating medical procedures. In the field of anesthesiology, infection control includes protection of anesthesia workers and the patients from infection, and the prevention of contamination of all anesthesia devices, especially the anesthesia machine. In maxillofacial surgery, anesthesia management has some unique characteristics because the procedures in maxillofacial surgery are aerosol-forming, resulting in the resulting in the aerosols dispersing into the environment. It is considered that the global effects of the coronavirus disease 2019 infection will continue for longer. Thus, the anesthesiologist working in the oral and maxillofacial surgery operating rooms should closely follow the current guidelines regarding the pandemic and take the necessary precautions to protect patients and staff from the risk of infection. Clinicians should provide their anesthesia precautions and management program during COVİD-19 pandemic for general anesthesia settings in oral and maxillofacial surgery.

## Introduction

Coronavirus affects both humans and animals as novel pathogens. The recent coronavirus was determined after pneumonia cases in Wuhan at the end of 2019. It rapidly resulted in an epidemic throughout China and spread over the world causing a pandemic. The coronavirus disease 2019 has been defined as COVID-19 in February 2020 by the World Health Organization (WHO).^1^ The coronavirus pathogen was found in the same subgenus with the severe acute respiratory syndrome virus (SARS) as a beta coronavirus within a different clade such that the angiotensin-converting enzyme 2 (ACE2) receptors used by the coronavirus for entering cells were similar to the SARS coronavirus.^2^ The Coronavirus Study Group of the International Committee on Taxonomy of Viruses has determined that this virus is designated as a severe acute respiratory syndrome coronavirus 2 (SARS-CoV-2).^3^ The Middle East respiratory syndrome (MERS) was another coronavirus.^4,5^

The virus has spread to nearly all countries in the world and affected people in terms of economic, social, psychological, and public health. It is thought that the virus was transmitted from animal to humans initially in Wuhan, and after that began the person-to-person transmission by the route of respiratory tract.^6^ Direct transmission from person to person replicates SARS-CoV-2 and is thought to occur through close-range contact between people. The virus transmission can occur via droplets that are thought to typically travel about 2 m (6 feet) while speaking, coughing, or sneezing. In addition, it can be transmitted by direct contact from an infected surface to the eyes, nose, or mouth. In dentistry, a close physical relationship with the patient, coming into contact with saliva and blood, and frequent aerosol production are the main risk factors for virus transmission. Furthermore, many viruses can easily be transmitted by saliva.^7-9^ Thus, this easy transmission feature makes it difficult to fight the virus, especially in social environments and hospitals. The type and duration of exposure and the use of preventive measures affect the transmission of SARS-CoV-2 infection.^9^ Severe acute respiratory syndrome coronavirus 2 was shown to contaminate room surfaces in hospital rooms of COVID-19 patients.^11,12^ Several international organizations and professional societies have issued guidelines or recommendations for perioperative care during the COVID-19 pandemic, which are based on expert opinion.^13-16^

In the field of anesthesiology, infection control includes protection of anaesthesia workers and the patients from infection, and the prevention of contamination of all anesthesia devices, especially the anesthesia machine. Infection control precautions have been recommended by the American Society of Anesthesiologists (ASA).^17^ Anesthesia management requires a specific approach in dentistry and maxillofacial surgery. Nearly all anesthetic procedures including tracheal intubation, non-invasive ventilation, tracheotomy, cardiopulmonary resuscitation, manual Balloon-mask ventilation before the intubation, and bronchoscopy in the operating rooms are aerosol-generating medical procedures. Maxillofacial surgeons work on the oral cavity, similar to that of anesthesiologists during the procedure, and they can easily inhale aerosols from the air aerosol. Thus, those working in the field of maxillofacial surgery (anesthesiologists and surgeons) are at risk of infection of both COVID-19 and other respiratory infection sources. Therefore, personal protective equipment (PPE) usage is necessary for the prevention and transmission of COVID-19 in every environment, that is, an operating room.^18^ In fact, in oral and maxillofacial surgery, procedures are performed intraorally resulting in aerosol, which is a risk for the patient under sedation in the operating room.^18^ Improving protective strategies for aerosol-generating surgery procedures is important for operating room providers. For the protection of personnel, the WHO and Centers for Disease Control and Prevention (CDC) guidance suggest to minimize the number of personnel in the room, to use appropriate PPE, and to avoid high amounts of aerosol producing procedures.^19,20^ However, beside this, hospitals must develop their self-local plans for aerosol-generating medical procedures in suspected/confirmed COVID-19 patients. The CDC has also reported a number of recommendations for emergency cases, as well as elective cases.^20^

During the COVID-19 pandemic, we determined a local management policy for all procedures and also general anesthesia settings based on legislation and scientific data. To avoid the undesirable effects of the COVID-19 pandemic, a well preparation and process management are required. Emergency cases that cannot be delayed may undergo surgery under general anesthesia or sedation protocols. The definition of the concept of “emergency” is generally variable and dependent on the surgeon’s desicion. Therefore, the anesthesiologist, and the surgeon should act together as a team to make the ideal and correct decision for operation of the patient. It can be suggested that sedation would be a better choice for patients than general anesthesia in the pandemic era because, it does not require an intubation procedure that includes a tube insertion from outside to the lung. In fact, in oral and maxillofacial operations, procedures are performed intraorally which result in an aerosol generation, which is a risk for the person in the operating room under sedation. So, PPE becomes more important for the team.

## Management of the Preoperative Period

What should we consider in the preoperative period in maxillofacial surgery patients prior to anesthesia during COVID-19 pandemic? Certainly, preoperative evaluation is a vital consideration. The anesthesiologists are responsible to answer following questions firstly, during preoperative patient evaluation. Is general anesthesia indicated? Is the general anesthesia application in this patient correct based on the current WHO and ASA (American Society of Anethesiologists) recommendations? Is the surgery necessary or urgent? Can the surgery be delayed to a time that includes less risk of infection? If surgery is mandatory, does the medical condition of the patient appropriate for the procedure?

For avoiding transmission of COVID-19 infection, PPE such as gloves, masks, respirators, goggles, gowns, coveralls and face shield usage has become indispensable for all settings in hospitals to protect healthcare workers.^21^ Accepting every patient as if they are COVID-19 positive and, approaching them cautiously should be the basic principle. Thus, taking maximum safety precautions would be an ideal approach to prevent the transmission and spread of infection in the pandemic era. The ASA reported on the essential laboratory tests that must be evaluated before surgery. According to ASA guidelines, lung radiography or electrocardiogram is not mandatory for patients of ASA I classification due to the stable medical condition of patients.^22^ However, the COVID-19 pandemic has brought innovations in clinical settings and preoperative evaluation. COVİD-19 diagnosis is performed by a polymerase chain reaction (PCR) test, which is routinely recommended 48 hours before the surgery.^23^ The PCR test is known to be reliable if it is positive, but negative results do not exclude COVID-19 because of the false-negative rate in nasopharyngeal swabs being 17-63%.^23^

A detailed history should be obtained from the patients including all preoperative evaluations. Initially, the anesthesiologist should consider the patient’s history of infection, whether he had COVID-19, or recently had contact with a COVID-19 patient. Furthermore, the anesthesiologist should also investigate whether there is an allergy or a family history associated with anesthesia in previous anesthesia applications. A preoperative examination should be performed with PPE using gloves, surgical or N95 mask, eye protection visor, and a disposable surgical bonnet by an anesthesiologist. After body temperature is measured, the patient should be taken to the examination room alone to minimize person-to-person contact. If the body temperature measured is above 37.3^°^C, the patient must be directed to the infection clinic. Detailed physical examinations should be conducted and lung auscultation findings should be investigated carefully. Laboratory results must be checked to see whether any abnormality is present. Before and after the examination, the anesthesiologist should disinfect the hands by washing for 20 seconds with tap water and soap or via an appropriate disinfectant. In addition, floors and all surfaces should be cleaned and disinfected with 2-3% hydrogen peroxide after the examination.^24^

A negative PCR test result does not mean the danger of infection has passed. Caution should be continued and precautions should never be disrupted. Therefore, clinicians must follow the current guidelines published by world renowned organizations. Rapid tests can be used for determining COVID-19 but it is not recommended for an initial COVID-19 diagnosis due to their low sensitivity.^25^ Sometimes a PCR test is negative but the lung image results support a diagnosis of COVID-19 in patients.^26^ Accordingly, lung radiography and auscultation findings of patients are important in preoperative evaluation.

In maxillofacial surgery, airway examination and evaluation of the patients are essential due to the type of surgery. Especially in temporomandibular joint diseases, limited joint movement also restricts mouth opening which makes intubation difficult; thus, it should be determined whether difficult intubation is expected in the patient. Airway management is often provided by fiberoptic bronchoscopy, which is suggested as the gold standard method in limited mouth opening situations. In addition, temporomandibular joint surgeries sometimes require tracheotomy attempts for providing a safe airway during perioperative period due to the inability of airway management. Therefore, fiberoptic bronchoscopy and tracheotomy procedures require more attention in the COVID-19 pandemic era due to them being aerosol-generating invasive procedures. Endotracheal intubation, extubation, bronchoscopy, tracheotomy, aspiration, mask ventilation, and noninvasive mechanical ventilation are aerosol-generating procedures, where all of them are performed in maxillofacial surgery operating rooms during general anesthesia applications.^27^ The anesthesiologists have a higher risk than other healthcare providers due to managing the airway and ventilation.^24^ Therefore, safe infection prevention protocols must be taken for the anesthesiologist during the perioperative period for confirmed or suspected COVID-19 patients in maxillofacial surgery.

### Preparation and Planning of the Operating Room

Standard prevention measures against droplet-transmitted infections should be taken for all operating room staff. These preventions include universal rules such as handwashing with soap or hygiene with 2-3% hydrogen peroxide, PPE (gloves, mask, and goggles), equipment disinfection and cleaning, surface disinfection (with 2-3% hydrogen peroxide or 75% alcohol).^24^ Minimizing the number of staff in the operating room during anesthesiology settings and during surgery is recommended to decrease transmission risk. The prevention protocol should begin with patient transport. Both the direct laryngoscopes and fiberoptic bronchoscopy equipment must be disinfected in the maxillofacial surgery operating room. For airway management or surgery procedures, the operating theatres should be established as negative pressure rooms and labeled as “airborne infection isolation rooms” with a minimum of 12 air changes per hour.^28^ A high-efficiency particulate air filter is recommended to prevent infection.^29,30^ A 30-minute waiting period is recommended after intubation and extubation so that the room air can be changed 12 times per hour.^30^

### Preparation for Emergency Operations

Emergency cases are generally maxillofacial trauma patients in oral and maxillofacial surgery. The traumatic damage to the maxillofacial region can cause difficulty in intubation at a higher rate than in the normal population. than the normal population. Emergency airway management processes require caution. Before transporting the patient to the operating room, the body temperature must be measured and then anesthesiologists must obtain the medical history of the patient. A detailed physical examination must be performed and the chest computed tomography and/or chest x-ray must be seen. In suspected or confirmed COVID-19 situations, nonemergency surgical procedures should be delayed. Patients should be taken into the isolation holding area before the surgery and then transferred to the operating room.^24^ A surgical mask should be used on patients, and health care workers should use PPE during transport.

## Airway Management

### Preparation

Evaluation of the difficult airway should be performed in preoperative examination. Emergency airway management equipment for managing difficult intubation and drugs should be prepared before the surgery in the operating room. Health departments and the anesthesiology department should adopt appropriate PPE. Disposable equipment would be better for procedures of anesthesiology if it is possible.^30,31^ As a recommendation, during the outbreak, adequate equipment, PPE, and medications must be stored in hospitals.^32^ The number of anesthesiology workers and the number of persons in the operating theatre should be decreased. The intubation should be performed by the most experienced anesthesiologist and there must be an assistant to help with the procedure. If a difficult airway is suspected and advanced methods will be required, a second experienced physician, wearing PPE, should also be available. An emergency airway bag or cart preferably including single-use equipment should be held at the anteroom to manage emergency airway situations.^30^

Low-flow nasal oxygen therapy (flows <5 L/min) is recommended during intubation to avoid the risk of hypoxia. A viral filter must be connected to the mask. Also, an heat and moisture exchange filter must be placed between the facemask and the elbow connector, making it as close as possible to the patient.^33,34^ Preoxygenation during 5 minutes and minimum gas flow (≤6L O_2_ minute^−1^) applications are recommended. The anesthetic circuit is suggested to use a circle circuit or the Mapleson C circuit. A tight face mask for a short period of time can be used during mask ventilation.^31,33^

### Intubation Procedure

Video laryngoscopes are recommended during the outbreak to minimize failed intubation.^35^ Maxillofacial surgery usually requires nasotracheal intubation. Nasotracheal intubation is different from oral intubation procedure and requires experience in this area. Nasal procedures such as endoscopy have been determined as a significant risk factor in terms of COVID-19 due to high viral titer in the nasal cavity.^36^ The nasal intubation procedure similarly has a high risk. After anesthesia induction, rapid sequence intubation must be done with 1.5 mg/kg rocuronium for muscle relaxation. Antisialogogic agents would be useful to decrease secretion and viral transmission. To avoid oxygen desaturation during mask ventilation, manual ventilation support with small tidal volumes should be ensured.^30^ The other important information is that mechanical ventilation should not be started before the ventilation circuit connection, and during disconnection, the ETT (Endotracheal Intubation) should be clamped.^37^ Fiberoptic intubation in a fully paralyzed patient may be safer for aerosol generation. Nevertheless, equipment cleaning after the procedure is important.

A tracheotomy procedure may be needed as a preventive procedure after the surgery or may be required due to failed intubation. During the tracheotomy procedure, adequate paralysis and limiting the use of suctioning during the procedure are recommended in order to minimize aerosol exposure. A closed in-line suction unit is suggested to be used.^38^

## Anesthesia Maintenance and Postoperative Period Management

Appropriate anesthesia management should be decided before the surgery according to the patient’s medical condition and surgical requirements. Moderate sedation, amnesia, and an adequate muscle relaxant should be provided in order to prevent couch. Ventilation parameters should be set carefully to avoid lung damage.

At the end of the surgery, extubation time must be decided according to the patient’s ventilation parameters; before the extubation, a good aspiration of the oral cavity and nasopharynx will be better. A normal saline wet gauze may cover the nose and mouth before the intubation and after the extubation during mask ventilation for decreasing aerosol generation. Extubation can be made under a transparent cover. If needed, positive pressure ventilation can be provided during this period. When the patient’s consciousness is restored, the patients are taken to preferably negative pressure or isolated rooms.^39^ Waiting for the full recovery of the patient in the operating room is better than using the recovery room to avoid transmission. During transport, the patient should wear a surgical mask, and the transfer team should be with their PPE. Using high-efficiency filters on both inspiratory and expiratory limbs would be a clever approach. Filters should be changed when the surgery duration lasts more than 4 hours. If the patient will be transported intubated, a filter should be added to the endotracheal tube. The anesthesia machine, monitors, and pumps should all be covered by packs with the availability of a control panel. Carbon dioxide absorbents should be replaced after every case.^41^ Postoperative patients who do not need an intensive care unit should be awakened in the operating room. When the patient is ready to go to the inpatient service, the road to the room should be isolated or the ICU should be cleaned again.^41^ The anesthesia machine should be carefully disinfected according to disinfection rules. All anesthesia drugs and equipment must be used for only one patient and must be disposed after use. The patient must be managed according to the infection management department’s recommendation in the inpatient service.

## Postoperative Pain Management

In maxillofacial surgery, different postoperative analgesia approaches may be required depending on the type of surgery. Postoperative pain is an acute clinical pain for which the use for multimodal analgesia is often suggested. There are multiple medications with different mechanisms of action. The side effects of the agents used in postoperative pain are also different.^42^ Currently, multimodal analgesia has an increased role in the post-COVID era. As a part of multimodal analgesia, acetaminophen or paracetamol is suggested to be applied for mild-to-moderate pain alone or in combination with nonsteroidal anti-inflammatory drugs (NSAIDs).^43^ Paracetamol is a safe agent during the COVID era and useful for fever, headache, and pain, but its liver toxicity must not be forgotten.^44^ Nonsteroidal anti-inflammatory drugs are effective for mild-to-moderate pain also but are relatively contraindicated in respiratory disorders. Nonsteroidal anti-inflammatory drugs increase susceptibility to the COVID-19 virus by upregulating entry through the ACE2 receptors. Although this information is not fully adequate, it is nonetheless important in the COVID-19 process.^44^ After oral and maxillofacial surgeries, paracetamol alone does not adequately solve pain problems every time, sometimes NSAIDs are needed to be used together for postoperative pain management. Therefore, there is no strong evidence for avoiding NSAIDs in all patients diagnosed with COVID-19. Thus, clinicians must decide on using NSAIDs according to patients’ condition.^45,46^

Opioids have a complex effect on the immune system caused by the type of opioid, dose, and the patient’s condition of natural immunity. Although opioids are immunosuppressive, pain itself also causes immunosuppression.^47-49^ Accordingly, opioid usage requires caution in the COVID-19 pandemic. Corticosteroids, which can be used in pain management, are often preferred for decreasing postoperative edemas in oral and maxillofacial surgery. Corticosteroids can increase infection risk because of immuno-suppressant features.^49^ Care should be taken when deciding on corticosteroid administration during the COVID period, considering the dose to be administered.

The COVID-19 pandemic is predicted to affect the global population for longer period. Although elective surgeries are recommended to be postponed to a later date, emergency surgeries sometimes become necessary. Anesthesia management has become more challenging during the COVID-19 pandemic due to aerosol-generating procedures in the oral and maxillofacial surgery area. Anesthesiologists working in oral and maxillofacial surgery operating rooms should closely follow the current guidelines regarding the pandemic and take the necessary precautions to protect the patient and staff from the risk of infection and thus the harmful effects of COVID-19. Clinicians should apply their anesthesia management program during the COVID-19 pandemic for general anesthesia settings in oral and maxillofacial surgery. This program must include strategies of a detailed preoperative evaluation, good operating room preparation, safety airway management procedure for the patients and anesthesia providers, well-designed anesthesia maintenance, and postoperative period management. Anesthesiologists must know the patients’ preoperative COVID-19 status and must plan the anesthesia method according to recent guidelines recommendations about oral and maxillofacial surgery.
